# THE EFFECT OF INCOME SECURITY IN OLD AGE ON HEALTH OUTCOMES: A COMPARISON BETWEEN CHINA AND THE US

**DOI:** 10.1093/geroni/igad104.1151

**Published:** 2023-12-21

**Authors:** Heng Wu

**Affiliations:** Dalhousie University, Halifax, Nova Scotia, Canada

## Abstract

This paper examines the social determinants of health, particularly the social, political, and economic context of socioeconomic status and health outcomes. Many studies on the political economy of health have highlighted the effects of welfare states on health and health inequalities, typically using welfare state regimes as proxies for social policies. Few research studies have described the association between frailty phenotype and financial wellbeing, however, particularly considering the three-legged stool of retirement income security (public pensions, private pensions, and personal savings/assets) across different welfare state regimes. This study investigates the effects of old-age income disparities on frailty among older adults in the United States and China. Specifically, this paper examines the associations between different sources of old-age income (public and private pensions, personal savings and assets, earnings, workplace subsidies, worker’s compensation, household subsidies, and unemployment insurance benefits) and the five-item frailty phenotype from the RAND Health and Retirement Study (HRS) and the China Health and Retirement Longitudinal Study (CHARLS). The findings reveal that health outcomes measured by the Fried’s frailty phenotype vary considerably by country and income type.

